# Global diversity and genetic landscape of natural populations and hatchery stocks of largemouth bass *micropterus salmoides* across American and Asian regions

**DOI:** 10.1038/s41598-019-53026-3

**Published:** 2019-11-13

**Authors:** Dan Wang, Hong Yao, Yan-He Li, Yong-Jiang Xu, Xu-Fa Ma, Han-Ping Wang

**Affiliations:** 1 Aquatic Genetics and Breeding Laboratory, The Ohio State University South Centers, Piketon, Ohio, USA; 2 Yangtze River Fisheries Research Institute, Chinese Academy of Fishery Sciences, Wuhan, Hubei China; 3 College of Fisheries, Key Lab of Agricultural Animal Genetics, Breeding and Reproduction of Ministry of Education, Huazhong Agricultural University, Wuhan, Hubei China

**Keywords:** Biodiversity, Population genetics

## Abstract

Although largemouth bass *Micropterus salmoides* has shown its extremely economic, ecological, and aquacultural significances throughout the North American and Asian continents, systematic evaluation of genetic variation and structure of wild and cultured populations of the species is yet to be documented. In this study, we investigated the genetic structure of *M. salmoides* from 20 wild populations and five cultured stocks across the United States and China using eight microsatellite loci, which are standard genetic markers for population genetic analysis. Our major findings are as follows: (1) the result of *Fst* showed largemouth bass had high genetic differentiation, and the gene flow indicated the genetic exchange among wild populations is difficult; (2) AMOVA showed that 14.05% of the variation was among populations, and 85.95% of the variation was within populations; (3) The majority of largemouth bass populations had a significant heterozygosity excess, which is likely to indicate a previous population bottleneck; (4) Allelic richness was lower among cultured populations than among wild populations; (5) Effective population size in hatcheries could promote high levels of genetic variation among individuals and minimize loss of genetic diversity; China’s largemouth bass originated from northern largemouth bass of USA. The information provides valuable basis for development of appropriate conservation policies for fisheries and aquaculture genetic breeding programs in largemouth bass.

## Introduction

Largemouth bass *Micropterus salmoides* (Lacépède) is native to South-eastern United States^1–3^. As one of the top predatory species, it has shown its extremely economic and ecological significances throughout the North American Continent^4–8^. This freshwater bass had gained the loyalty of the estimated 50 million U.S. anglers at the beginning of this century^9^; thus, fingerlings and juveniles of this species are widely cultivated in public and private hatcheries to supply recreational fishing waters^10^. It has also been regarded as one of the dominated non-salmonid freshwater food fishes^11^.

It is universally acknowledged that the divergence of lineages within largemouth bass occurred due to geological changes during some geologic time^12–14^; and two subspecies of this fish species, i.e. the Florida largemouth bass *M. s. floridanus* and the northern largemouth bass *M. s. salmoides*, occupied their native habitats from peninsular “Florida to throughout northeastern Mexico”, southeastern Canada, and the U.S. corridor in between^15^. The further divergence of the latter subspecies at northern and southern latitudes has been identified through genetic analysis^16–19^. Growth differences and environmental adaptability of these populations has been focused on by many researchers^20^; and the perceived superior growth characteristics of *M. s. floridanus* resulted in its fast and wide introduction to far north of its native range almost all over the United States^21,22^.

Largemouth bass with uncertain lineages have also been introduced to many countries in Europe^23–26^, Asia^27–29^, and Africa^1,30^ since the 19th century and gained access to a large area of habitats outside its native range; and it is playing greater role in freshwater fisheries and ecological systems of these regions.

A trio of factors, including arbitrary introduction, intraspecific hybrid, and environmental stress, makes it difficult to detect the impacts of human activity, especially aquaculture and recreational fishing, on the genetic diversity of largemouth bass. Philipp revealed the genetic distinctions among some largemouth bass groups were continually being diluted by introgression^16^. Studies on four cultured populations of largemouth bass in Guangdong, China indicated that the stocks are northern largemouth bass depleted in genetic variation^28^. Actually, short-term allelic changes like this had been a concern for some populations within United States decades ago^31–33^. Recently, attention has been paid to allele persistence of largemouth bass. Johnson and Fulton finished comparison of the allele frequencies of three discriminate allozyme loci between Florida and northern largemouth bass and inferred that genes persisted through many generations^20^. What puzzles ecologists and culturists who are working with largemouth bass is that they are not sure about the taxonomy and genetic background when they are confronted with either a wild or hatchery produced largemouth bass population.

Microsatellite loci are standard genetic markers for population genetic analysis, and have many advantages, including codominant alleles, extensive genome coverage (present in both coding and noncoding regions), high allelic diversity at the loci, and the ability to determine genotypes from small samples of tissue using polymerase chain reaction (PCR)^34^. Although single nucleotide polymorphisms (SNPs) are becoming new tools in genetics and genomics studies, comparative analyses of SNP and microsatellite marker data for population genetic analysis in *Diabrotica virgifera virgifera* resulted in similar conclusions with respect to population structure^35^. Therefore, microsatellite should still be an invaluable tool for various population genetics studies^36^.

Lutz-Carrillo^37^ examined microsatellite DNA variation at 11 loci in populations of Florida largemouth bass *M. s. floridanus* and northern largemouth bass *M. s. salmoides* from northern and southern latitudes in North America and found despite the systematic introduction of Florida largemouth bass into Texas, genetic integrity of a number of populations had not been or were only minimally affected. But they suggested that a full assessment of the variation throughout the range of each subspecies should be conducted to identify minimally impacted environments and quantify the levels of gene flow from impacted to intact water bodies to continue to restart the necessary work performed by Philipp^16^ over 20 years ago. Lutz-Carrillo subsequently developed 52 novel microsatellite markers from *M. s. floridanus*, for use in population genetic studies and expected them to be helpful for the resolution of hatchery-released individuals in the wild, introgression among *Micropterus* species, and gene flow following stocking and restoration efforts^38^.

In this study, we investigated the population genetic structure of hatchery stocks and wild populations in the largemouth bass based on eight polymorphic microsatellite loci. Samples encompassed both populations from wild habitats and hatcheries or domestic fish ponds. Our objectives were as follows: (1) to determine genetic diversity of wild largemouth bass populations in North America; (2) to evaluate genetic variation in hatchery largemouth bass and identify differential strain contribution affecting the genetic variability (3) to provide valuable information for the fishery management and conservation implication of largemouth bass.

## Results

### Genetic diversity

Across all 25 populations sampled, the w96 locus had the highest number of alleles (18.8) while the mdo6 locus had the least (6.6). The total number of alleles at each locus ranged from 3 to 53. The average number of alleles across all loci was the highest (20.8) in the RBRMS and lowest (7.3) in the SJFL. No significant genetic associations among loci were observed. The value of observed heterozygosities per population was detected from 0.0213 to 1. The expected heterozygosities were from 0.1158 to 0.9905 (Table 1).

**Table 1:** Summary statistics for 8 microsatellites loci among 25 largemouth bass collection.

Locus		Location												
		GZCH	LSOH	Q8TX	KMFL	SJFL	PLWI	DRTX	LKTX	PRMS	CLMS	BORMS	RBRMS	BR2SC
lar 7	*n*	50	50	30	50	50	48	38	50	50	50	34	50	14
	*Na*	5	6	13	16	5	8	8	6	7	10	11	13	10
	*H*_*O*_	0.2200	0.1400	0.6667	0.5000	0.6800	0.0833	0.5526	0.4600	0.1000	0.2000	0.5294	0.4800	0.6429
	*H*_*E*_	0.3683	0.2240	0.8638	0.7869	0.9307	0.2667	0.6554	0.4602	0.1897	0.3836	0.6400	0.5414	0.7143
	*P*_*HW*_	0.0001**	0.0018*	0.0338	0.0000**	0.0000**	0.0000**	0.1754	0.0848	0.0000**	0.0000**	0.0863	0.0143	0.5374
mdo 6	*n*	50	50	30	50	50	48	38	50	50	50	34	50	14
	*Na*	4	6	5	5	5	4	4	4	4	7	7	12	9
	*H*_*O*_	0.5000	0.1800	0.1667	0.0400	0.1800	0.0625	0.5000	0.5200	0.3400	0.3600	0.2647	0.5000	0.7857
	*H*_*E*_	0.5042	0.2238	0.6102	0.7030	0.7265	0.1599	0.5028	0.5176	0.3618	0.4814	0.3446	0.7354	0.8862
	*P*_*HW*_	0.3519	0.0121	0.0000**	0.0000**	0.0000**	0.0000**	0.0010**	0.8638	0.0009**	0.0000**	0.0050*	0.0000**	0.0357
w157	*n*	50	50	30	50	50	48	38	50	50	50	34	50	14
	*Na*	11	25	11	13	9	11	11	8	11	16	13	15	15
	*H*_*O*_	0.5000	0.2800	0.6000	0.7200	0.6600	0.4593	0.8947	0.4800	0.8800	0.5600	0.5294	0.7600	0.6429
	*H*_*E*_	0.7020	0.8790	0.7537	0.9067	0.9103	0.7338	0.8309	0.5788	0.8374	0.8824	0.7309	0.8531	0.9550
	*P*_*HW*_	0.0000**	0.0000**	0.0092	0.0000**	0.0000**	0.0000**	0.1130	0.0359	0.0573	0.0000**	0.0023*	0.0809	0.0042*
w13	*n*	50	50	7	50	8	7	38	50	50	50	34	50	14
	*Na*	8	12	7	—	6	14	14	21	16	29	21	20	15
	*H*_*O*_	0.7000	0.8800	0.8571	—	0.6250	0.4286	0.9211	0.8600	0.8800	0.9000	0.7353	0.8800	0.8571
	*H*_*E*_	0.6293	0.9244	0.9011	—	0.9667	0.8022	0.9242	0.9281	0.8923	0.9739	0.9254	0.9067	0.9577
	*P*_*HW*_	0.0024*	0.0860	0.7215	—	0.0034*	0.0012**	0.0101	0.0809	0.0018*	0.0106	0.0005**	0.0972	0.1892
w134	*n*	50	50	30	50	50	48	38	50	50	50	34	50	14
	*Na*	5	4	12	10	4	5	5	4	12	14	12	23	11
	*H*_*O*_	0.6600	0.4600	0.9333	0.9400	0.7600	0.4375	0.5000	0.1000	0.7600	0.8400	0.6177	0.7800	0.7857
	*H*_*E*_	0.5008	0.5269	0.8695	0.8410	0.8715	0.6629	0.5712	0.1158	0.8693	0.9279	0.8709	0.9434	0.8968
	*P*_*HW*_	0.0816	0.0019*	0.1598	0.0000**	0.0001**	0.0000**	0.2578	0.0151	0.0000**	0.0000**	0.0000**	0.0000**	0.2452
w190	*n*	50	50	30	50	50	48	38	50	50	50	34	50	14
	*Na*	13	11	9	9	14	7	7	10	31	25	18	11	12
	*H*_*O*_	0.9800	0.9800	0.7333	0.7200	0.6800	0.4167	0.5263	0.7200	0.6800	0.8200	0.2059	0.1400	0.1429
	*H*_*E*_	0.8016	0.8198	0.8396	0.8240	0.8741	0.8421	0.5312	0.6042	0.9659	0.9459	0.8644	0.9091	0.9339
	*P*_*HW*_	0.0001**	0.0218	0.0414	0.0016*	0.0000**	0.0000**	0.0575	0.0000**	0.0000**	0.0011**	0.0004**	0.0000**	0.0000**
w90	*n*	50	50	30	50	50	48	38	50	50	50	34	50	14
	*Na*	8	9	9	13	8	6	6	31	27	28	22	19	14
	*H*_*O*_	1.000	0.9400	0.8667	0.9000	0.6400	0.5000	0.7105	0.8400	0.8600	0.9200	0.7941	0.7600	0.7857
	*H*_*E*_	0.7348	0.7125	0.8548	0.8513	0.8127	0.8083	0.7067	0.9647	0.9360	0.9665	0.9513	0.9535	0.9577
	*P*_*HW*_	0.0000**	0.0000**	0.0827	0.0000**	0.0000**	0.0000**	0.0616	0.0000**	0.0000**	0.0000**	0.0000**	0.0000**	0.0007**
w96	*n*	50	50	30	50	50	9	38	50	50	50	34	50	14
	*Na*	16	23	12	14	7	15	15	19	20	35	27	53	17
	*H*_*O*_	0.6800	0.8800	0.3333	0.2600	0.3400	0.2222	1.0000	0.7800	0.8800	0.8800	0.7647	0.8400	0.7143
	*H*_*E*_	0.8580	0.9372	0.8859	0.9234	0.8679	0.9281	0.9263	0.9053	0.9453	0.9667	0.9666	0.9905	0.9656
	*P*_*HW*_	0.0000**	0.1075	0.0000**	0.0000**	0.0000**	0.0000**	0.5098	0.0055*	0.0000**	0.0000**	0.0017*	0.0000**	0.0000**
**Locus**		**Location**												
		**CRSC**	**THMS**	**NHMS**	**BR3SC**	**SLMN**	**HLMN**	**NLOH**	**ALOH**	**NROH**	**ACLOH**	**MSOH**	**PKOH**	
lar 7	*n*	29	50	50	11	42	43	50	47	42	45	50	22	
	*Na*	10	17	3	8	5	3	4	6	6	9	8	5	
	*H*_*O*_	0.4138	0.8200	0.3200	0.8182	0.1191	0.1395	0.0400	0.0213	0.0952	0.1778	0.1000	0.2727	
	*H*_*E*_	0.5433	0.8885	0.3284	0.6970	0.1595	0.1335	0.3550	0.4935	0.6804	0.6392	0.6055	0.6765	
	*P*_*HW*_	0.0207	0.0007**	0.0097	0.8823	0.0409	1.0000	0.0000**	0.0000**	0.0000**	0.0000**	0.0000**	0.0000**	
mdo 6	*n*	29	50	50	11	42	43	50	47	42	45	50	22	
	*Na*	10	10	4	7	5	4	9	11	6	9	8	6	
	*H*_*O*_	0.5517	0.1600	0.0600	0.7273	0.1905	0.1395	0.2600	0.4681	0.0714	0.0889	0.0600	0.3636	
	*H*_*E*_	0.7653	0.6481	0.4697	0.8918	0.6779	0.3658	0.6414	0.8307	0.5548	0.7189	0.6263	0.6480	
	*P*_*HW*_	0.0001**	0.0000**	0.0000**	0.1355	0.0000**	0.0000**	0.0000**	0.0000**	0.0000**	0.0000**	0.0000**	0.0006	
w157	*n*	29	50	50	11	42	28	50	47	42	45	50	22	
	*Na*	10	21	13	9	16	12	16	16	18	27	18	11	
	*H*_*O*_	0.8621	0.9000	0.4800	0.5455	0.2857	0.1071	0.6400	0.6170	0.5000	0.7333	0.3800	0.3636	
	*H*_*E*_	0.7326	0.9224	0.8798	0.8485	0.9157	0.8942	0.8855	0.9426	0.8942	0.9388	0.9323	0.9239	
	*P*_*HW*_	0.1981	0.0250	0.0000**	0.0085	0.0000**	0.0000**	0.0000**	0.0000**	0.0000**	0.0000**	0.0000**	0.0000**	
w13	*n*	29	50	50	11	42	43	50	47	42	45	50	22	
	*Na*	9	20	18	11	14	13	35	36	18	35	17	20	
	*H*_*O*_	0.5862	0.6600	0.8000	0.7273	0.5000	0.2558	0.9600	0.7660	0.5476	0.6889	0.8200	0.7727	
	*H*_*E*_	0.6745	0.9184	0.9509	0.9524	0.7742	0.8249	0.9804	0.9794	0.9343	0.9718	0.9531	0.9767	
	*P*_*HW*_	0.2893	0.0000**	0.0000**	0.0000**	0.0000**	0.0000**	0.0010**	0.0000**	0.0000**	0.0145	0.0000**	0.0002**	
w134	*n*	29	50	50	11	42	43	50	47	42	45	50	22	
	*Na*	16	19	5	6	8	10	5	8	7	10	6	4	
	*H*_*O*_	0.9310	0.7600	0.4000	0.8182	0.6429	0.8140	0.4400	0.2128	0.1429	0.2889	0.2800	0.4091	
	*H*_*E*_	0.7967	0.8584	0.4735	0.8831	0.5772	0.7880	0.7065	0.8067	0.7625	0.8082	0.6622	0.7135	
	*P*_*HW*_	0.6120	0.0000**	0.0017*	0.0285	0.0000**	0.0000**	0.0000**	0.0000**	0.0000**	0.0000**	0.0000**	0.0004**	
w190	*n*	29	50	50	11	42	38	50	47	42	45	50	22	
	*Na*	13	23	8	8	10	18	18	23	17	23	8	11	
	*H*_*O*_	0.5517	0.6600	0.6800	0.5455	0.5000	0.5526	0.5200	0.5319	0.3571	0.6667	0.5600	0.6818	
	*H*_*E*_	0.8554	0.8503	0.7535	0.9134	0.7932	0.8667	0.8768	0.9094	0.8887	0.8864	0.5541	0.9154	
	*P*_*HW*_	0.0000**	0.0000**	0.0000**	0.0011**	0.0000**	0.0000**	0.0000**	0.0000**	0.0000**	0.0000**	0.0259	0.0000**	
w90	*n*	29	50	50	11	42	20	50	47	42	45	50	22	
	*Na*	23	25	5	8	14	14	9	10	10	13	7	7	
	*H*_*O*_	0.9310	0.8400	0.1400	0.5455	0.4762	0.9500	0.2600	0.5957	0.5238	0.6667	0.6600	0.3182	
	*H*_*E*_	0.9528	0.8978	0.2739	0.9004	0.7283	0.9090	0.6537	0.8200	0.8310	0.9101	0.6467	0.8066	
	*P*_*HW*_	0.1369	0.0000**	0.0002**	0.0011**	0.0000**	0.0012*	0.0000**	0.0000**	0.0000**	0.0000**	0.0154	0.0000**	
w96	*n*	29	50	50	11	42	31	50	47	42	45	50	22	
	*Na*	12	24	10	13	11	18	15	17	18	29	13	16	
	*H*_*O*_	0.4828	0.7200	0.5600	0.5455	0.6667	0.8387	0.6400	0.7447	0.6429	0.7333	0.6600	0.7727	
	*H*_*E*_	0.7810	0.9150	0.7261	0.9004	0.7381	0.9492	0.7461	0.8520	0.8893	0.9171	0.8202	0.8710	
	*P*_*HW*_	0.0000**	0.0004**	0.0006*	0.0000**	0.0456	0.0000**	0.0000**	0.0000**	0.0000**	0.0004**	0.0000**	0.0227	

*n*: sample size; *Na*: observed number of alleles; *H*_*O*_: observed heterozygosity; *H*_*E*_:expected heterozygosity; *P*_*HW*_: probability in Hardy-Weinberg equilibrium. Denoted significant departure from HWE after Bonferroni correction (*P < 0.00625, **P < 0.00125).

The test for fit to Hardy-Weinberg proportions revealed that most of loci were found to be deviated from Hardy-Weinberg because of heterozygote deficiencies or excess (after bonferroni correction).

### Gene flow

The result of *Fst* was 0.19 (among 25 populations), showing that largemouth bass had high genetic differentiation, and the gene flow indicated the genetic exchange among wild populations is difficult (Table 2).

**Table 2:** Summary of F-Statistics and gene flow for all loci among 25 populations.

Locus	Fis	Fit	Fst	Nm
lar7	0.3401	0.4456	0.1599	1.3132
mdo6	0.4772	0.6282	0.2889	0.6152
w157	0.2770	0.4000	0.1701	1.2200
w13	0.2462	0.3878	0.1878	1.0811
w134	0.1914	0.3250	0.1652	1.2629
w190	0.4591	0.5784	0.2206	0.8833
w90	0.1449	0.2511	0.1242	0.7622
w96	0.4219	0.5195	0.1688	1.2312
Mean	0.3039	0.4351	0.1885	1.0762

Nm = Gene flow estimated from Fst = 0.25(1 − Fst)/Fst.

### Genetic variation

Genetic variation statistics obtained by AMOVA were significant at all hierarchical levels. When all populations were grouped together and all loci were considered, 14.05% of the variation was among populations, and 85.95% of the variation was within populations (Table 3).

**Table 3:** AMOVA analysis results for 25 populations of largemouth bass.

Source of variation	Sum of squares	Variance components	Percentage of variation
Among populations	885.83	0.41Va	14.05%
Within populations	5212.39	2.52Vb	85.95%
Total	6098.22	2.93	

### Population structure

**Figure 1:**
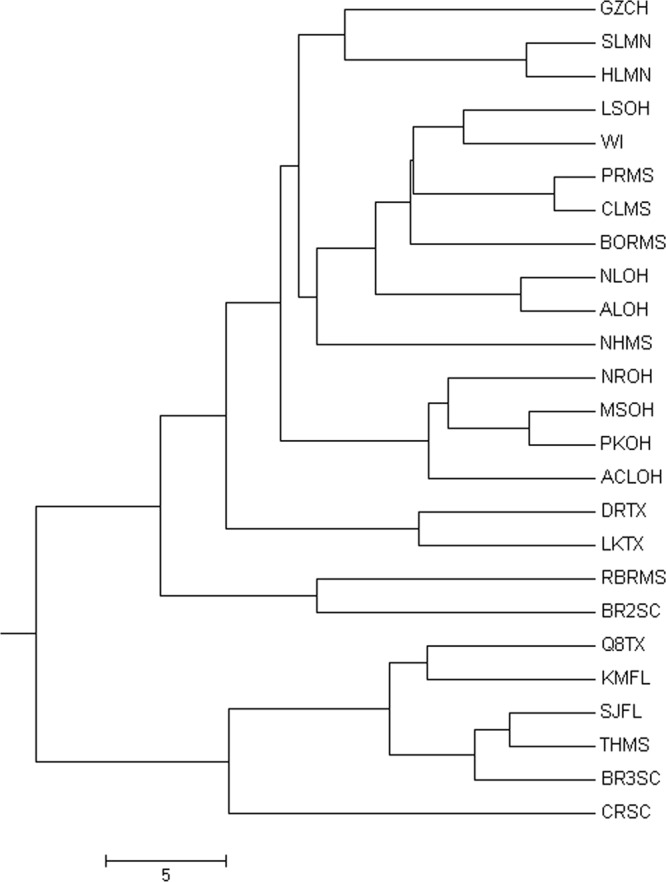
UPGMA tree of 25 populations based on the allele-sharing distance.

The UPGMA tree built from the matrix of pairwise allele-sharing distance among 25 populations (Fig. 1) and the assignment tests revealed the multilocus microsatellite genotypes to discriminate populations of largemouth bass. The phylogeny based on eight microsatellites revealed a clear distinction between northern and southern populations, although samples from 25 populations were different.

**Figure 2:**
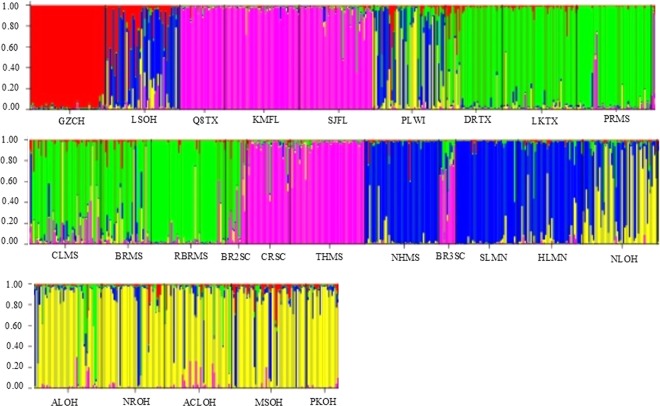
Structure analysis of populations (the inferred clusters, k = 4). The sampling location abbreviations (see Table 4) are indicated along the X-axis. Each vertical line represents one individual, and Y-coordinate denotes each individual’s percentage assignment to each of these seven genetic clusters.

The software STRUCTURE was used to examine how the sampled populations clustered based on the genetic data. The preliminary STRUCTURE run indicated that the most likely number of clusters was two; thus, the second and more robust simulation was run with k = 26. However, in the latter analysis, the results suggested that the most likely number of clusters was four (Fig. 2).

### Population bottlenecks

The majority of largemouth bass populations had a significant heterozygosity excess, which is likely to indicate a previous population bottleneck. However, under the SMM and TPM models, there is no bottleneck signature; under the IAM model, PLWI (*P* = 0.01) and LKTX (*P* = 0.008) suggested that there had been a recent population bottleneck.

## Discussions

This study is the first to use microsatellite DNA markers to examine the genetic variation within and among wild and hatchery populations of largemouth bass across America and China. Other freshwater fish species using microsatellites revealed thenumber of alleles per locus (5.4)^39^ was lower than we observed (18.1), which may have been due to the geographic we sampled, the number of populations we sampled, or the loci we selected. Some loci failed to be amplified in cultured populations, which might be due to high sensitivity of these loci under environmental pressure and selective breeding. The highest values of the mean number of alleles and private alleles were detected in the RBRMS population. In the past, hatchery procedures often had overlooked genetic differences between populations, as well as the consequences of reducing heterogeneity through inbreeding^16^. In the present study, the value of observed heterozygosities per population was detected from 0.0213 to 1. The expected heterozygosities were from 0.1158 to 0.9905. Thus, the populations we collected can provide a wide range of selection to avoid reducing heterogeneity resulting from inbreeding. The inspection of genetic information provides fishery managers with a means for understanding and predicting stocking population variation for regulations and other management programs in largemouth bass fisheries.

Genetic diversity is increasingly declining as a result of various factors, including direct human impact and structural alteration of ecosystems resultingfrom changes in human life styles, bottleneck experienced, inbreeding, etc^40^. Genetic diversity values are a better indicator of the genetic polymorphism within a population, because estimates of the mean number of alleles are influenced by sample size. In the present study, the majority of largemouth bass populations had a significant heterozygosity excess, which is likely to indicate a previous population bottleneck. However, comparing Southern and Northern populations of largemouth bass, the South Carolina populations showed higher values of gene diversity and allele richness. The populations have maintained their genetic integrity and been only minimally affected. Nevertheless, the high genetic diversity of invasive species may be caused by hybridization and variation. In this study, the results showed that the largemouth bass had high genetic differentiation. In addition, according to AMOVA analysis, overall genetic differentiation among largemouth bass from the 25 populations was significant (*P* < 0.05), suggesting significant genetic differentiation among localities or populations. However, overall, the genetic variations within populations are higher than those among populations. In the natural environment, the long-term success of stocked largemouth bass might be the greatest for cohorts with the highest levels of genetic variability^16^. Microsatellite allelic frequencies and *Fst* analyses showed that all 25 populations were genetically unique. These genetic differences have resulted from long-term exposure to natural selective pressures and provide thespecies with enough variation to deal with the Barge range of different environmental conditions in which it exists^41^.

The largemouth bass has two subspecies, the Florida largemouth bass *M. s. floridanus* and the northern largemouth bass *M. s. salmoides*. They occupy their native habitats from peninsular Florida to throughout northeastern Mexico, southeastern Canada, and the U.S. corridor in between^15^. The genetic difference is particularly pronounced for the two recognized subspecies, but pertains to populations within each subspecies as well. The UPGMA tree built from the matrix of pairwise allele-sharing distance among 25 populations across America and the assignment tests revealed the multilocus microsatellite genotypes to discriminate populations of largemouth bass. The phylogeny based on eight microsatellites revealed a clear distinction between northern and southern populations. The China population and Minneapolis populations were clustered in a group, indicating that China’s largemouth bass originated fromnorthern largemouth bass, since China introduced largemouth bassonly one time from California, USA before our sampling time.

In conclusion, there is relatively high genetic diversity of wild largemouth bass populations in North America, and significant genetic differentiation among localities or populations in the largemouth bass, but obviously lower genetic diversity in hatchery stocks. The study suggested that China’s largemouth bass originated from northern largemouth bass of USA. This study provided valuable information for the fishery management and conservation implication of largemouth bass, and indicated that the differentiation in largemouth bass was caused by the founder effect. Besides the founder effect, geographicaldistance might also be responsible for the significant genetic differentiation among wild populations. These analyses clearly reveal substantial genetic differences among populations in the United States. This study demonstrates the need for incorporating genetic information and principles into current and future fisheries management programs. However, the effectiveness of the stocking populations should be evaluated and the long-term effects on the genetic composition of specified largemouth bass populations should be monitored.

## Methods

### Populations studied

**Figure 3:**
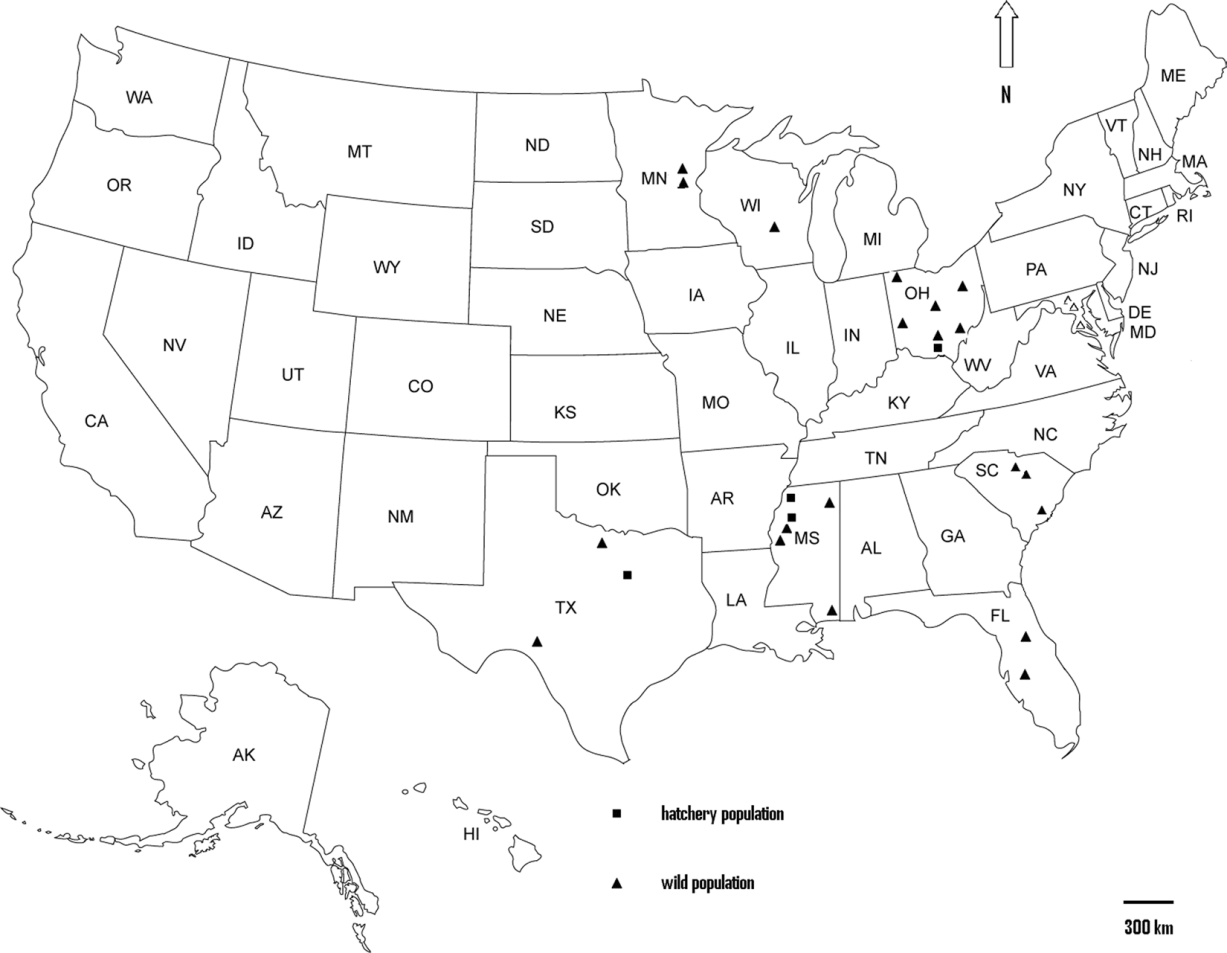
Sampling sites for 20 wild and 5 hatchery *M. salmoides* populations. Abbreviations are as listed in Table 4.

A total of 1,045 individuals were collected by trapping and/or electro-fishing from 25 different localities and coordinated by geographic sampling coordinates throughout North America and China (Fig. 1 and Supplementary Table S1). Twenty wild populations and five hatchery populations were analyzed. Details of the samples are given in Table 4, and the approximate geographical location of the sampled populations is indicated in Fig. 3.

**Table 4:** The information of populations, state/country and population’s abbreviation for samples collected.

Populations	State/Country	Abbreviation
**Wild populations**		
Lake Snowden	OH	LSOH
Nettle Lake	OH	NLOH
Acton Lake	OH	ACLOH
North Reservoir	OH	NROH
Alum Creek lake	OH	ACLOH
Mary Street	OH	MSOH
Pike Lake	WI	PLWI
Spirit Lake	MN	SLMN
Hill Lake	MN	HLMN
Q8	TX	Q8TX
Devils Rivers	TX	DRTX
Pascagoula River	MS	PRMS
Columbus Lake	MS	CLMS
Belzoni Old River	MS	BRMS
Ross Barnett Reservoir	MS	RBRMS
Broad River Reach 2	SC	BR2SC
Broad River Reach 3	SC	BR3SC
Copper River	SC	CRSC
Q9 Kissimme	FL	KMFL
St. Johns	FL	SJFL
**Hatchery populations**		
Guangzhou	CHINA	GZCH
Lake Kickapoo	TX	LKTX
Turcotle Fish Hatchery	MS	THMS
North MC Fish Hatchery	MS	NHMS
Piketon	OH	PKOH

Fin clips were the sources of nuclear DNA. They were collected from the freshly caught fish and immediately preserved in 95% ethanol.

### Extraction of DNA and microsatellite amplification

Total DNA was extracted from an optimum volume of fin tissue following a modified version of the pure gene protocol for extraction from fish tissue^37^. Amplification of microsatellite loci were performed using eight primers selected from those isolated and characterized by Lutz-Carrillo (Table 5)^16,37,38^. Each locus was amplified with a three-primer system in which only the M13 and CAG primers were fluorescently labeled with FAM, HEX, or NED.

**Table 5:** Characteristics of largemouth bass microsatellite markers.

Locus	Motif (s)	Allele Size Range (bp)	Tm (°C)	References
lar 7	(GATA)4	121–193	47	Lutz-Carrillo *et al*.^37^
mdo 6	(CA)7(TA)4	160–176	55	Lutz-Carrillo *et al*.^37^
w13	(ACAT)6	528–572	60	Lutz-Carrillo *et al*.^28^
w90	(ACAT)6	164–233	55	Lutz-Carrillo *et al*.^28^
w96	(AGAT)15 + (AGAC)6	345–581	60	Lutz-Carrillo *et al*.^28^
w134	(AC)9	154–170	57	Lutz-Carrillo *et al*.^28^
w157	(AC)21	169–202	60	Lutz-Carrillo *et al*.^28^
w190	(AC)7 + (AC)22	228–286	60	Lutz-Carrillo *et al*.^28^

Polymerase chain reaction of 6 μL contains 3 μL of JumpStart RedMix (Sigma), 1.5 pmol of both non-tailed and labelled primers, and 0.1 pmol of the tailed primer, 25 ng DNA, in the presence of 100 μm spermidine. Amplification was be conducted in PTC-100 thermal cyclers (MJ Research) using an initial denaturation at 94 °C for 2 min, followed by 35 cycles of 30 s denaturation at 94 °C, 30 s annealing at a locus-specific temperature (Table 5), 30 s extension at 72 °C, and a final 5-min extension at 72 °C. Amplification products were separated using an ABI 3130 Prism DNA genetic analyzer.

### Data analyses

#### Analyses of genetic variation and genetic differentiation

Deviations from Hardy-Weinberg equilibrium (HWE) and linkage disequilibrium (LD) were being tested for all locus-population and locus-locus combinations using ARLEQUIN version 2.0^42^. Significance levels were be modulated for multiple comparisons using the sequential Bonferroni method^43^. Genetic variation was evaluated according to observed heterozygosity and the number of alleles per locus within populations and regions. Private alleles among populations were computed using GenAlEx 6^44^.

#### Analyses of molecular variance

Analyses of molecular variance (AMOVA) were conducted with Arlequin. One AMOVA was performed under the null hypothesis of no genetic structure. AMOVAS will examine population structure after combining populations in various ways to test for geographical structure.

#### Assignment tests

Genetic structure among populations was analyzed based on a chord distance according to Cavalli-Sforza and Edwards^45^. A UPGMAtree was constructed using the programs NEIGHBOR and CONSENSE in PHYLIP version 3.6 in terms of Felsenstein^46^. Genetic structure among individuals was assessed using the model-based Bayesian clustering method according to Pritchard^47^ and Lutz-Carrillo^37^. Assignment tests were performed with geneclass version 2.0^48^. The program STRUCTURE was used to identify genetic clusters without using any prior information of the sampling location of the individuals^47^. Two separate analyses were carried out in STRCTURE, both times with allele frequencies as correlated and the admixture model. The first analysis was used to infer the most likely range for K. Here, we used a burn-in of 10000 and a MCMC length of 5000 iterations and the simulated number of populations from K = 1–25. The upper limit of 25 was chosen as this corresponds to the number of sampled populations. Twelve independent simulations were performed of each K to check for consistency across runs. The preliminary results were assessed using the Evanno method where the most likely K was determined by the distribution of △K.

#### Bottlenecks analyses

Allelic richness was calculated with FSTAT version 2.9.3.2^49^. All these tests were adjusted for multiple simultaneous comparisons using a sequential Bonferroni correction. Means are reported as ±SE. We used bottleneck version 1.2.02^50^ to test for evidence consistent with recent population bottlenecks or expansions by recognizing significant heterozygosity excess or deficiency for each population using a Wilcoxon sign–rank test. We conducted the tests using 1,000 iterations using the SMM of microsatellite evolution, as well as a two-phased mutation model (TPM). Allele frequency data was tested for evidence of a “heterozygosity excess” (HE) using the program bottleneck^48,50^. Three statistical tests (sign test, standardized differences test, and Wilcoxen signed-ranks test) were conducted in order to determine whether there is significant HE, which may indicate that a recent bottleneck has occurred.

#### Ethics

This study and all experimental procedures involving animals were performed according to the protocol approved by the Ohio State University Institutional Animal Care and Use Committee. Permissions for field studies were granted by: Ohio Department of Natural Resources, South Carolina Department of Natural Resources, Texas Parks & Wildlife Department, Minnesota Department of Natural Resources, Mississippi Wildlife Fisheries & Parks, Florida Blackwater Fisheries Research and Development Center, Wisconsin Genoa Federal Fish Hatchery (Supplementary Table S1).

## Supplementary information

Supplementary Table S1
