# Supplementary material for: Global diversity and genetic landscape of natural populations and hatchery stocks of largemouth bass micropterus salmoides across American and Asian regions
Source: Sci Rep. 2019 Nov 13;9:16697. doi: 10.1038/s41598-019-53026-3 (PMC6853949; doi:10.1038/s41598-019-53026-3)
Supplement: Supplementary file 1 — Supplementary Table S1 [file 41598_2019_53026_MOESM1_ESM.doc]

**Global diversity and genetic landscape of natural populations and hatchery stocks of largemouth bass *micropterus salmoides* across American and Asian regions**

Dan Wang^1,2,3^, Hong Yao^1^, Yanhe Li^1,3^, Yongjiang Xu^1^, Xufa Ma^3^, and Hanping Wang^1*^

^1^Fish Genetics and Breeding Laboratory, The Ohio State University South Centers, Ohio, United State

^2^Yangtze River Fisheries Research Institute, Chinese Academy of Fishery Sciences, Wuhan, Hubei, China

^3^College of Fisheries, Key Lab of Agricultural Animal Genetics, Breeding and Reproduction of Ministry of Education, Huazhong Agricultural University, Wuhan, Hubei, China

E-mail: [wang.900@osu.edu](mailto:wang.900@osu.edu)

Work telephone Number: 740-289-2071

Work fax number: 740-289-4591

**Supplementary Table S1. Geographic sampling coordinates of each site**

| **Populations** | **State/Country** | | **Sampling Coordinates** | | |
| --- | --- | --- | --- | --- | --- |
| **Wild populations** | |  | |  |  |
| Lake Snowden | | OH | | Ohio Department of Natural Resources (Scott Hale and Richard Zweifel) |  |
| Nettle Lake | | OH | |  |
| Acton Lake | | OH | |  |
| North Reservoir | | OH | |  |
| Alum Creek lake | | OH | |  |
| Mary Street | | OH | |  |
| Pike Lake | | WI | | Wisconsin Genoa Federal Fish Hatchery (Doug Aloisi) |  |
|  | |  | |  |  |
| Spirit Lake | | MN | | Minnesota Department of Natural Resources |  |
| Hill Lake | | MN | |  |
|  | |  | |  |  |
| Q8 | | TX | | [Texas Parks & Wildlife Department](https://www.google.com/url?sa=t&rct=j&q=&esrc=s&frm=1&source=web&cd=1&sqi=2&ved=0CB4QFjAA&url=https%3A%2F%2Ftpwd.texas.gov%2F&ei=u9vUVMLmFMSWyAT8kIHwBw&usg=AFQjCNFbsBoYhiiyVyUKFQ1O3vNfRL4Qrg&bvm=bv.85464276,d.aWw) (Dijar Lutz) |  |
| Devils Rivers | | TX | |  |
|  | |  | |  |  |
| Pascagoula River | | MS | | Mississippi Wildlife Fisheries & Parks |  |
| Columbus Lake | | MS | |  |
| Belzoni Old River | | MS | |  |
| Ross Barnett Reservoir | | MS | |  |
| Broad River Reach 2 | | SC | | South Carolina Department of Natural Resources (James Bulak) |  |
| Broad River Reach 3 | | SC | |  |
| Copper River | | SC | |  |
| Kissimme | | FL | | Florida Blackwater Fisheries Research and Development Center (David Yeager) |  |
| St. Johns | | FL | |  |
| **Hatchery populations** | |  | |  |  |
| Guangzhou | | CHINA | | Pear River Fishery Research Institute (Junjie Bai and Shengjie Li) |  |
|  | |  | |  |  |
| Lake Kickapoo | | TX | | [Texas Parks & Wildlife Department](https://www.google.com/url?sa=t&rct=j&q=&esrc=s&frm=1&source=web&cd=1&sqi=2&ved=0CB4QFjAA&url=https%3A%2F%2Ftpwd.texas.gov%2F&ei=u9vUVMLmFMSWyAT8kIHwBw&usg=AFQjCNFbsBoYhiiyVyUKFQ1O3vNfRL4Qrg&bvm=bv.85464276,d.aWw) (Dijar Lutz) |  |
| Turcotle Fish Hatchery | | MS | | Mississippi Wildlife Fisheries & Parks |  |
| North MC Fish Hatchery | | MS | |  |
|  | |  | |  |  |
| Piketon | | OH | | OSU South Centers (Dean Rapp and Paul O’Bryant) |  |
